# Delay of transfer from the intensive care unit: a prospective observational study of incidence, causes, and financial impact

**DOI:** 10.1186/cc12807

**Published:** 2013-07-04

**Authors:** Daniel W Johnson, Ulrich H Schmidt, Edward A Bittner, Benjamin Christensen, Retsef Levi, Richard M Pino

**Affiliations:** 1 Department of Anesthesiology, University of Nebraska Medical Center, 984455 Nebraska Medical Center, Omaha, NE 68198-4455, USA; 2 Department of Anesthesia, Critical Care & Pain Medicine, Massachusetts General Hospital, Harvard Medical School, 55 Fruit St, Boston, MA 02114, USA; 3 Sloan School of Management, Massachusetts Institute of Technology, 50 Memorial Dr, Cambridge, MA 02142, USA

**Keywords:** critical care utilization, cost analysis, triage, resource allocation, organizational efficiency, workflow

## Abstract

**Introduction:**

A paucity of literature exists regarding delays in transfer out of the intensive care unit. We sought to analyze the incidence, causes, and costs of delayed transfer from a surgical intensive care unit (SICU).

**Methods:**

An IRB-approved prospective observational study was conducted from January 24, 2010, to July 31, 2010, of all 731 patients transferred from a 20-bed SICU at a large tertiary-care academic medical center. Data were collected on patients who were medically ready for transfer to the floor who remained in the SICU for at least 1 extra day. Reasons for delay were examined, and extra costs associated were estimated.

**Results:**

Transfer to the floor was delayed in 22% (*n *= 160) of the 731 patients transferred from the SICU. Delays ranged from 1 to 6 days (mean, 1.5 days; median, 2 days). The extra costs associated with delays were estimated to be $581,790 during the study period, or $21,547 per week. The most common reasons for delay in transfer were lack of available surgical-floor bed (71% (114 of 160)), lack of room appropriate for infectious contact precautions (18% (28 of 160)), change of primary service (Surgery to Medicine) (7% (11 of 160)), and lack of available patient attendant ("sitter" for mildly delirious patients) (3% (five of 160)). A positive association was found between the daily hospital census and the daily number of SICU beds occupied by patients delayed in transfer (Spearman rho = 0.27; *P *< 0.0001).

**Conclusions:**

Delay in transfer from the SICU is common and costly. The most common reason for delay is insufficient availability of surgical-floor beds. Delay in transfer is associated with high hospital census. Further study of this problem is necessary.

## Introduction

In recent years, increasing attention has been paid to the sizable and growing costs of critical care services. The percentage of the United States' gross domestic product used for critical care services increased from the year 2000 to 2005 by 13.7%, from 0.58% to 0.66% [1]. Optimal use of intensive care unit (ICU) resources is an important goal for individual hospitals and healthcare systems and is an essential component of the effort to contain healthcare expenditures. At our institution, we found that many patients who met criteria for transfer out of the ICU remained in the ICU for a longer time than necessary. We investigated the incidence of delayed transfer of surgical ICU patients and sought to determine the causes for delays and to estimate the costs associated with them. We hypothesized that transfers out of our ICU are delayed by 1 or more days at least 20% of the time, and that the majority of delays are due to a lack of available floor beds.

## Materials and methods

### Setting

After approval by the hospital's Institutional Review Board, the study was conducted in the Surgical Intensive Care Unit (SICU) of the Massachusetts General Hospital from January 24, 2010, to July 31, 2010. Informed consent was not necessary because of the observational nature of the study. Patient care was not affected by the study, and individual patient information was not used during the analysis of data. MGH is a 900-bed university-affiliated tertiary care center with 1.5 million outpatient visits and 47,000 inpatient admissions annually. The hospital is a level 1 trauma center. The SICU is a 20-bed unit with >1,400 annual admissions, primarily from the complex trauma, vascular, thoracic, and general surgical populations. It is staffed 24 hours per day by attending intensivists in addition to critical care fellows and residents.

### Transfer process

A patient was classified as ready for transfer to the floor by consensus among the patient's intensivist, surgeon, and SICU nurse, based on guidelines for discharge criteria, with emphasis on hemodynamic and respiratory stability. After a patient was deemed ready for transfer, a request to the admissions office was placed to locate an appropriate floor bed. Hospital-based nursing triage supervisors were aware of the bed availability throughout the hospital and supported the process. When a bed became available, the patient was transferred out of the SICU.

### Reasons for delay

Before the study, we surveyed intensivists, surgeons, SICU nurses, nursing triage supervisors, and administrators to determine the perceived reasons for transfer delay. Based on these responses, we predefined the following four reasons: lack of available surgical-floor bed, lack of an appropriate bed for infectious contact precautions, change of primary service (Surgery to Medicine), and lack of available patient attendant ("sitter" for mildly delirious patients).

### Demographic and clinical factors

All patients who were admitted to the SICU during the study period were included in the study, without regard for demographic or clinical factors.

### Data collection and definition of delay

During the study period, a SICU intensivist (DWJ) contacted the SICU charge nurse each night to identify those patients who were delayed in transfer. Patients were classified as delayed in transfer when the intensivist, surgeon, and SICU nurse were in agreement that the patient could be transferred to the floor, yet the patient remained in the SICU past 00:01 that night. The intensivist and charge nurse discussed each case to determine which of the four reasons (or "other") best characterized the patient's reason for delay. Duration of ICU stay, duration of hospital stay, admitting surgical service, destination (type of surgical floor), date deemed ready for transfer, date/time of actual transfer, and daily hospital census were recorded throughout the study period.

### Cost analysis

We used the difference between the 2005 national average cost of a day in the ICU ($3,518) and a day in a non-ICU hospital bed ($1,153) to estimate the extra costs associated with patients remaining in the SICU unnecessarily [1]. This difference ($2,365) was multiplied by the total number of delay days (246) to generate the estimated total costs of the delays.

### Statistics

All data analysis was performed by using Stata 10 (Stata-Corp LP, College Station, TX, USA). Continuous variables with a normal distribution are expressed as mean and standard deviation (SD). Ordinal variables are expressed as median and range. The χ^2 ^test was used to compare absolute numbers and proportions. The Spearman rho was used to evaluate the association between the daily hospital census and the daily number of SICU beds occupied by patients delayed in transfer.

## Results

**Figure 1:**
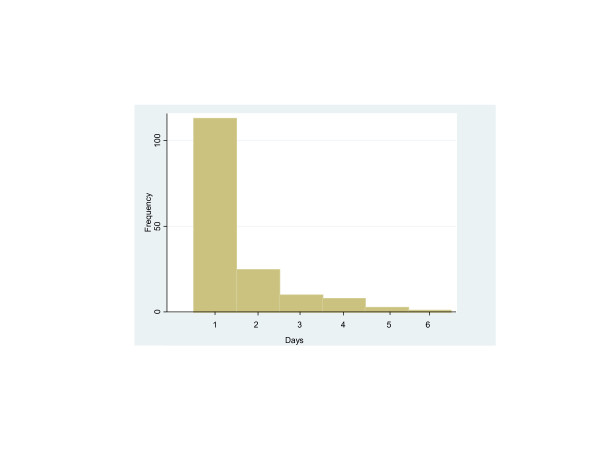
**Distribution of length of delay in intensive care unit (ICU) transfer (in days)**.

During the study period, 731 patients were transferred from the SICU to the floor. Of these patients, 160 (22%) experienced a delay in transfer of at least 1 day. The delays in transfer ranged from 1 to 6 days (mean, 1.5 days; median, 2 days; Figure 1). The most common reason for delay was lack of availability of a surgical-floor bed (71% (114 of 160)). The lack of an appropriate room for infectious contact precautions accounted for 18% (28 of 160) of delays. The remaining causes were change of primary service (Surgery to Medicine) at 7% (11 of 160), and lack of available patient attendant ("sitter" for mildly delirious patients) at 3% (five of 160).

The cost associated with delays in transfer was estimated to be $581,790 for the entire study period, or $21,547 per week.

The daily hospital census was positively correlated with the number of SICU beds occupied by delayed patients (Spearman rho = 0.27; *P *< 0.0001). Delayed patients were significantly more likely than nondelayed patients to be transferred during night shifts, between 19:00 to 06:59, (21% (33 of 160) versus 12% (67 of 571); χ^2 ^= 10.6; *P *< 0.005).

## Discussion

In this study, we found that delay in transfer from the SICU occurred in 22% of cases, with lack of availability of surgical-floor beds as the most common reason, accounting for 71% of delayed transfers. The ICU beds in our hospital constitute approximately 15% of the total number of beds. Although the absolute numbers and ratios of ICU beds-to-floor beds vary from institution to institution, our hospital's ratio of 15% ICU-to-total hospital beds is equal to the national average of 15% [1]. Recent studies describe similar incidence and financial impacts [3,4]. Our results are similar to those from a 2004 study in an Australian combined medical-surgical ICU that found that 27% of ICU transfers were delayed by at least 8 hours, and that 81% of these delays were due to lack of available floor beds [5]. Whereas it is currently impossible to estimate the overall incidence of ICU-transfer delays in the United States and abroad, our study and these reports from other institutions suggest that the problem is widespread and contributes significantly to inefficiencies in healthcare systems.

ICU transfer delays have a large financial impact. Time spent in the ICU costs more than time spent on a regular floor, so delays in transfer naturally increase the overall cost of care. The cost difference between ICU days and floor days is driven mostly by the difference in staff-to-patient ratios in the ICU versus on the floor, including higher ratios of nurses, physicians, and therapists to patients.

The cost analysis in this study was performed by using data from the report of the United States critical care bed numbers, occupancy rates, payer mix, and costs published by Halpern and Pastores in 2010 [1]. By using national average costs, the results of this study are more generally applicable to the healthcare community at large.

A limitation of this method of cost analysis is that it assumes that ICU costs remain constant throughout the ICU stay. A study by Dasta and colleagues in 2005 [6] showed that costs are highest during the first 2 days of an ICU stay, and that higher costs are associated with mechanical ventilation. Patients who are stable and ready for discharge to the floor incur fewer costs than do critically ill patients. At our institution, the majority of daily ICU cost per patient is attributable to room and board, which includes nursing care. The daily cost of room and board in our ICU does not change after a patient is deemed ready for transfer to the floor, and the amount is not dramatically different from the estimated cost of an ICU day in the Halpern report; therefore, the cost-analysis model is valid for estimation of increased costs.

We analyzed the actual costs for delayed versus nondelayed patients (from the time each patient was deemed ready for transfer out of the ICU). Although actual patient-charge information is not publishable per our hospital's policies, the total additional costs of delays in transfer were greater than what would be predicted by the cost data in the Halpern report.

The increased costs associated with delayed transfer in our single-ICU study should prompt clinicians and administrators to examine closely ways to reduce or eliminate such delays. Preliminary results from a recent retrospective study showed similar increases in hospital costs associated with delayed transfer from the ICU [3]. Although the methods used in that study were different from ours, both studies came to the same conclusion: delays in transfer out of the ICU constitute a significant and costly problem.

A large number of delays were related to infectious contact precautions (28%). In accordance with Centers for Disease Control (CDC) guidelines, patients in our institution known to be infected or colonized with multidrug-resistant organisms or *Clostridium difficile *are required either to have single rooms or to share a room with patients with the same organism [7]. Providers caring for these patients are required to use contact precautions: gowns and gloves in addition to hand cleaning. A possible contribution to this element of the delay problem is that a majority of our hospital's non-ICU rooms are not private. Recently published studies appear to offer conflicting results regarding the efficacy of contact precautions; still, the recommendation to keep affected patients separated from unaffected patients is unlikely to change in the near future [8,9].

Delays in transfer were associated with high hospital census. At times during the study period, our hospital's census was >95% occupancy, and these times correlated with an increased number of SICU beds occupied by delayed patients. It has been shown that efficiency of acute care units is impaired when hospital occupancy rates exceed 85% [10]. Excessive hospital occupancy can lead to a bottleneck effect in which completely occupied floor beds prohibit transfers out of the ICU and thus prohibit the admission of critically ill surgical patients into the ICU. Our hospital is a level-1 trauma center and referral center for complex surgical operations. Prevention of SICU admissions because of excessively occupied floor beds results in trauma victims and critically ill surgical patients being admitted to nonsurgical ICUs.

Patients who were delayed in transfer were more likely than nondelayed patients to be transferred during night shifts. This is notable because of the previously published data that show that patients who are transferred at night have an increased likelihood of readmission to the ICU [11,12] and that patients who are readmitted to the ICU have an increased risk of hospital mortality [13]. The reason for the observed association between ICU transfer delay and nighttime transfer is not clear and requires further study.

Investigators recently reported a marked reduction in the incidence of delayed OR-to-SICU transfer after the implementation of measures aimed at facilitating early transfer of medically suitable patients out of the SICU [4]. In this study, the authors noted delays in OR-to-ICU transfer related to impeded SICU throughput and demonstrated a reduction in such delays after interventions reduced time to transfer out of the SICU. Similar delays occur in our institution, and they place a tremendous burden on OR personnel, surgeons, anesthesiologists, and OR administrators. The successful interventions by Young *et al. *[4] demonstrate that reduction in delayed transfer from the SICU can have a measurably positive impact on the hospital as a whole.

Possible contributions to the problem include the occupation of floor beds by patients ready to be discharged from the hospital, inefficiency in discharging patients from the ICU to home, and the subspecialization of surgical-floor beds. When a surgical floor is at capacity and discharge-ready patients remain in the floor's beds, transfers to the floor from the SICU are potentially delayed. Interventions to reduce the number of floor beds occupied by discharge-ready patients would likely reduce SICU-transfer delays.

Physicians and nurses in our ICU have relatively little experience in discharging patients home. Some of the patients who experienced long delays in transfer from the SICU to the floor were likely ready for discharge home. Efforts to improve recognition of discharge-ready patients and to educate staff members in the process of discharging home might be beneficial in reducing the unnecessary occupation of ICU beds. Patients in our hospital are transferred from the SICU to service-specific floors (for example, aortic surgery patients are transferred to a floor dedicated solely to vascular surgery). Only when clinically essential (for example, to create an available SICU bed for a patient with severe traumatic injuries) are patients transferred to floors other than their service-specific floor. De-specialization of surgical floors might reduce the incidence of delayed transfer from the SICU. The benefit of specialty surgical floors to patients, presumed by intensivists and surgeons at many large tertiary care centers, requires study for confirmation.

The discussion regarding the United States' need to curb its ever-increasing healthcare expenditures must include consideration of the costs associated with provision of critical care. Multiple reports have projected a shortfall of critical care services in the years to come [14,15]. Considering these projections and the budgetary constraints that the healthcare system faces, intensivists and hospital administrators must optimize the efficiency of each dollar spent in the ICU. In a system with too few ICU beds and inadequate financial resources, floor-ready patients occupying ICU beds represent a double-edged sword.

Although planning for the projected increased need for ICU beds seems prudent, improved utilization of existing ICU resources is an essential component of the strategic planning needed to address the problem. Reduction or elimination of delays in transfer from the ICU have the potential to increase ICU-bed capacity effectively without the physical creation of new ICU beds. Previous publications have discussed the importance of ICU outflow in overall ICU resource utilization, yet few studies of this problem have been conducted [16]. The paucity of literature addressing the problem in our study suggests that the issue has not been adequately quantified and analyzed. Further study of ICU-transfer delays, including studies of interventions to improve the problem, will likely improve patient care and resource utilization.

This study has several limitations. The study was performed in a single surgical ICU of a major academic medical center. Similar academic centers have reported delays in SICU transfer, but it is unknown whether such delays occur commonly in nonacademic settings. We used days as the time variable. It might be beneficial to use hours in future studies, more precisely to quantify the problem. Our cost analysis was based on national average costs of ICU and floor days. Future studies could collect and analyze cost data of the hospital or hospitals where the study is being conducted and account for the reduction in actual costs that occurs as patients' care needs lessen over time. Hospital census was used as a proxy for surgical-floor census. Future studies could analyze the actual census of destination floors for delayed ICU patients.

## Conclusions

Delay in transfer from the SICU is common and is associated with increased cost of hospitalization. Because the most common reason for this delay was insufficient availability of surgical-floor beds, future efforts should include emphasis on increasing the availability of floor beds.

## Key messages

• Delay in transfer from the SICU is common and costly.

• The most common reason for delay is insufficient availability of surgical-floor beds.

• Delay in transfer is associated with high hospital census.

## Abbreviations

CDC: Centers for Disease Control; ICU: intensive care unit; IRB: institutional review board; OR: operating room; SICU: surgical intensive care unit.

## Competing interests

The authors declare that they have no competing interests, financial or nonfinancial.

## Authors' contributions

DWJ designed the study, engaged in daily communication with ICU charge nurses, collected and recorded all data, analyzed all data, and drafted the preliminary, revised, and final versions of the manuscript. US provided input on the design of the study, assisted in designing the data-collection instrument, and edited the manuscript extensively. EAB provided input on the design of the study, performed all statistical analysis, created Figure 1, and edited the manuscript. BC and RL provided expertise on the financial elements of the study, conducted analysis of cost differences between delayed and nondelayed patients, and edited the manuscript. RMP conceived of the study, managed the IRB application, obtained IRB approval, provided input on the design of the study, and edited the manuscript. All authors read and approved the manuscript before submission.
